# Restricted Mean Survival Time—Can It Be a New Tool in Assessing the Survival of Non-Small Cell Lung Cancer Patients Treated with Immune Checkpoint Inhibitors?

**DOI:** 10.3390/diagnostics13111892

**Published:** 2023-05-29

**Authors:** Cristina-Florina Pîrlog, Raluca Costache, Andreea Ioana Paroșanu, Cristina Orlov Slavu, Mihaela Olaru, Ana Maria Popa, Cristian Iaciu, Irina Niță, Pompilia Moțatu, Horia Teodor Cotan, Alexandru Vlad Oprița, Daniel Costache, Loredana Sabina Cornelia Manolescu, Cornelia Nițipir

**Affiliations:** 1 Department of Oncology, Faculty of Medicine, “Carol Davila” University of Medicine and Pharmacy, 050474 Bucharest, Romania; 2 Department of Medical Oncology, Elias Emergency University Hospital, 011461 Bucharest, Romania; 3 Department of Internal Medicine and Gastroenterology, Faculty of Medicine, “Carol Davila” University of Medicine and Pharmacy, 050474 Bucharest, Romania; 4 Department of Medical Oncology, Monza Oncology Hospital, 013821 Bucharest, Romania; 5 Department of Medical Oncology, Municipal Hospital Ploiesti, 100409 Ploiesti, Romania; 6 Department of Medical Oncology, “Saint Nicholas” Hospital Pitești, 110124 Pitesti, Romania; 7 Third Department, Discipline Dermatology II, Faculty of Medicine, “Carol Davila” University of Medicine and Pharmacy, 050474 Bucharest, Romania; 8 Department of Microbiology, Parasitology and Virology, Faculty of Midwifery and Nursing, “Carol Davila” University of Medicine and Pharmacy, 050474 Bucharest, Romania; 9 Department of Virology, Institute of Virology “Stefan S. Nicolau”, 030304 Bucharest, Romania

**Keywords:** lung cancer, immune checkpoint inhibitors, immune-related adverse events, restricted mean survival time

## Abstract

Background: Lung cancer (LC) is the first and most lethal cancer in the world; identifying new methods to treat it, such as immune checkpoint inhibitors (ICIs), is needed. ICIs treatment is very effective, but it comes bundled with a series of immune-related adverse events (irAEs). Restricted mean survival time (RMST) is an alternative tool for assessing the patients’ survival when the proportional hazard assumption (PH) fails. Methods: We included in this analytical cross-sectional observational survey patients with metastatic non-small-cell lung cancer (NSCLC), treated for at least 6 months with ICIs in the first- and second-line settings. Using RMST, we estimated the overall survival (OS) of patients by dividing them into two groups. A multivariate Cox regression analysis was performed to determine the impact of the prognostic factors on OS. Results: Seventy-nine patients were included (68.4% men, mean age 63.8), and 34/79 (43%) presented irAEs. The OS RMST of the entire group was 30.91 months, with a survival median of 22 months. Thirty-two out of seventy-nine (40.5%) died before we ended our study. The OS RMST and death percentage favored the patients who presented irAEs (long-rank test, *p* = 0.036). The OS RMST of patients with irAEs was 35.7 months, with a number of deaths of 12/34 (35.29%), while the OS RMST of the patients without irAEs was 17 months, with a number of deaths of 20/45 (44.44%). The OS RMST by the line of treatment favored the first line of treatment. In this group, the presence of irAEs significantly impacted the survival of these patients (*p* = 0.0083). Moreover, patients that experienced low-grade irAEs had a better OS RMST. This result has to be cautiously regarded because of the small number of patients stratified according to the grades of irAEs. The prognostic factors for the survival were: the presence of irAEs, Eastern Cooperative Oncology Group (ECOG) performance status and the number of organs affected by metastasis. The risk of dying was 2.13 times higher for patients without irAEs than for the patients who presented irAEs, (CI) 95% of 1.03 to 4.39. Moreover, by increasing the ECOG performance status by one point, the risk of death increased by 2.28 times, with a CI 95% of 1.46 to 3.58, while the involvement of more metastatic organs was associated with a 1.60 times increase in the death risk, with a CI 95% of 1.09 to 2.36. Age and the type of tumor were not predictive for this analysis. Conclusions: The RMST is a new tool that helps researchers to better address the survival in studies with ICIs treatment where the PH fails, and the long-rank test is less efficient due to the existence of the long-term responses and delayed treatment effects. Patients with irAEs have a better prognosis than those without irAEs in the first-line settings. The ECOG performance status and the number of organs affected by metastasis must be considered when selecting patients for ICIs treatment.

## 1. Introduction

Lung cancer (LC) is the second most frequent type of cancer among men, with an incidence in Europe in 2020 of 14.8% and a mortality of 24.2% of all cancer types [1]. Although there are new modalities of diagnosis and treatment, the survival rate at 5 years, for all people and for all LC types is 22% [2].

In the last 6 years, the standard first-line treatment of NSCLC, without harboring mutations, included immunotherapy alone or the addition of platinum doublet chemotherapy (CHT) to immunotherapy. From 2015, when U.S. Food and Drug Administration (FDA) approved for the first time an immune checkpoint inhibitor (ICI) for treating advanced LC, until 2022, advances were made, and five ICIs there were approved in different lines in the treatment of LC.

The immune checkpoint inhibitors (ICIs) approved by the FDA are Imfinzi (Durvalumab) for the treatment of stage III unresectable NSCLC after definitive chemoradiotherapy (CHT-RT) [3]; Opvido (Nivolumab) in the second line treatment for advanced and metastatic NSCLC [4,5]; Tecentriq (Atezolizumab) in first the line treatment with Avastin (Bevacizumab); CHT (platinum doublet) and in further lines (second and third) alone for advanced and metastatic NSCLC [6,7]; Keytruda (Pembrolizumab) in first the line treatment either alone for metastatic NSCLC with programmed cell death ligand 1 (PD-L1) ≥50% or in combination with CHT for metastatic NSCLC regardless of PD-L1 expression, and in the second line settings in patients with tumor proportional score (TPS) ≥ 1% [8,9,10,11]; and Libtayo (Cemiplimab) as monotherapy in the treatment of advanced NSCLC with PD-L1 of at least 50% [12].

### 1.1. Immune-Related Adverse Events (irAEs)-Mechanism of APPEARANCE and Outcomes

Immune checkpoint inhibitors used in the treatment of LC are very efficient, but they come with secondary effects, such as the development of immune-related adverse events (irAEs).

There are several mechanisms implicated in the development of irAEs. The first one is the stimulation of the host’s own T cytotoxic lymphocytes to “fight” against cancer cells through ICIs usage. This stimulation directs the “fight” of T cytotoxic lymphocytes also to their own organs, resulting in an imbalance of immune homeostasis [13,14].

The second mechanism is represented by epitope spreading. ICIs therapy used in cancer leads to releasing in the bloodstream of self and non-self-antigens. These antigens are recognized by antigen-presenting cells (APC) and presented to T cells that develop new epitopes. These new epitopes allow T cells to attack not only tumor cells but also normal tissue cells [14,15].

The third mechanism is based on the production of antibodies with increased reactivity on non-self-antigens and decreased reactivity on self-antigens. The B cells activation contributes to irAEs pathways through increased cytokine production, antigen presentation to T cells and by increasing the secretion of autoreactive antibodies. Moreover, B cells can express programmed cell death protein 1 (PD-1) and programmed cell death ligand 1 (PD-L1) receptors that can be activated by ICIs therapy without the help of T cells [14].

The fourth mechanism implicated in the development of irAEs is direct molecular mimicry, which consists of the binding of anti-cytotoxic T-lymphocyte-associated protein 4 (CTLA-4) antibodies to the CTLA-4 proteins expressed in normal pituitary gland cells and triggering the complement cascade with the development of hypophysitis [13,14].

The fifth mechanism is related to the increased production of cytokines. The use of ICIs in cancer treatment may imbalance the tumor microenvironment toward inflammation and autoimmunity [14].

The sixth mechanism is related to the appearance of irAEs and is linked with the alteration of the gut microbiome. Some studies suggest that there is a relationship between the gut microbiome and the responses to irAEs treatment [14,16].

IrAEs are caused by ICIs therapy and can occur in any organ system. Dermatological toxicities appear first after the initiation of ICIs treatment; they are usually followed by gastrointestinal toxicities, and the last ones that appear are endocrine toxicities and hepatitis. These kinetics of the appearance of irAEs are common both to anti-CTLA-4, anti-PD-1 and PD-L1 therapy [17].

The appearance of irAEs depends on the agent we use and if we use it as a single agent or along with another one. In a study, Bai X. et al. concluded that the incidence of colitis, hepatobiliary disorders and pancreatitis were higher if anti-CTLA-4 were used and that polytherapy is a strong risk factor for developing those irAEs [18].

The management of irAEs depends on the degree of the reaction. Treatment for toxicity grades 1 and 2 consists in withholding immunotherapy, administering oral prednisone until the toxicity drops, and continuing the ICIs therapy afterward. Grades 3 and 4 of toxicity require permanent discontinuation of ICIs treatment and high doses of systemic steroids.

We know from the studies conducted on melanomas that the presence of irAEs is correlated with survival, and the onset of skin toxicity is a factor that predicts a better survival of patients [19,20]. In NSCLC, some studies confirm that the development of irAEs is correlated with survival [21], while other studies suggested that the development of irAEs is a predictor of poor survival. The study conducted by Suresh K. et al. confirmed that the development of pneumonitis decreases the survival rate in patients with NSCLC [22].

### 1.2. Restricted Mean Survival Time (RMST)

In order to analyze the treatment’s efficacity in randomized controlled trials (RCT) with time-to-event outcomes, it is necessary to determine the overall survival (OS) and progression-free survival (PFS). OS and PFS are the main endpoints in clinical trials. The analysis of the survival curves is performed using the Kaplan–Meier method and the long-rank test. To determine the effectiveness of the treatment, we can use the Cox proportional hazard. With the help of the Cox model, we can determine the hazard ratio (HR). The Cox regression model assumes that HR is constant over time, which means the existence of a proportional hazard (PH) [23,24].

In recent years, researchers have attempted to find other tools to evaluate OS for treatment with ICIs. The existence of long-term responses to treatment and delayed clinical effects led to the appearance of restricted mean survival time (RMST) [24,25].

RMST is a parameter that analyses the average survival time from 0 up to a specified point in time, and it reveals the area under the survival curve up to that point in time [26,27]. In the estimation of RMST, there is not necessary to have an existing PH. The RMST is a reliable endpoint in estimating survival when the PH assumption is violated [28,29].

This study aimed to determine whether the RMST can predict survival in patients with metastatic NSCLC treated with Nivolumab in the second line and Pembrolizumab in the first line. We presented here a detailed observational, cross-sectional study in which we studied the relationships between different impact factors such as irAEs, age, sex, ECOG, type of tumor, number of metastatic organs, type of treatment and the survival of patients with metastatic NSCLC.

## 2. Materials and Methods

### 2.1. Patients

We conducted an analytical cross-sectional observational survey on metastatic NSCLC patients treated in Elias University Emergency Hospital, Bucharest, Romania, between January 2018 and May 2022. Our research respects the guidelines and has the approval of the local Ethics Committee of the Elias University Emergency Hospital. The study procedures respected the ethical standards in the Helsinki Declaration. The protocol was approved by the Ethics Committee with the number 2171/18.03.2021. Seventy-nine patients with metastatic NSCLC were recruited for our study. The patients were followed up until 15 May 2022. They had at least 6 months of ICIs treatment either in the first line or in the second line.

### 2.2. The Inclusion Criteria

The inclusion criteria consisted of the following patients with metastatic NSCLC above 18 years old, with Eastern Cooperative Oncology Group (ECOG) status of 0, 1 and 2, treated with ICIs in first and second lines, without contraindications for treatment with ICIs and in the absence of pregnancy.

### 2.3. The Exclusion Criteria

**Figure 1:**
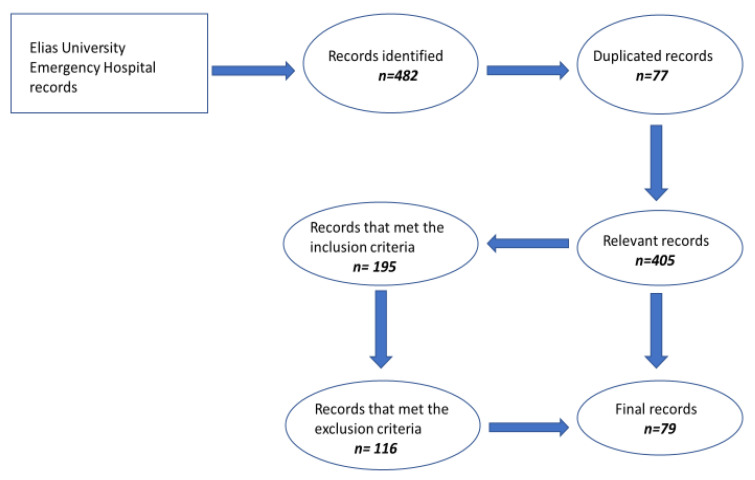
The screening process of the patients.

The exclusion criteria consisted of the following: patients with metastatic NSCLC with Eastern Cooperative Oncology Group (ECOG) performance status of 3 and 4, patients with autoimmune diseases, patients with positive infection with B or C hepatic viruses, patients with HIV-positive disease, patients treated with high doses of steroids, patients with incomplete dates for our database, patients with epidermal growth factor receptor (EGFR) mutation, patients with small cell lung cancer (SCLC), patients treated with ICIs in adjuvant stages, patients with no follow-up status and those who did not have at least 6 months of ICIs treatment, Figure 1.

Tumor staging was performed using the Classification of Malignant Tumors (TNM), and the patient’s evaluation was performed using the Immune-based Response Evaluation Criteria in Solid Tumors (iRECIST) criteria. Common Terminology Criteria for Adverse Events (CTCAE) v 5.0/November 2017 was used to classify the levels of toxicity.

### 2.4. Treatment

The patients’ treatment was comprised of Nivolumab administered at a fixed dose of 240 mg every 2 weeks (240 q2w), Pembrolizumab 200 mg every 3 weeks (200 mg q3w) alone or along with chemotherapy Paclitaxel (200 mg per square meter of body surface area) and Carboplatin (at a dose calculated to produce an area under the concentration–time curve of 6 mg per milliliter per minute) on day 1 every 3 weeks for 4 cycles, or Pemetrexed (500 mg per square meter) and either Cisplatin (75 mg per square meter of body-surface area) or Carboplatin (area under the concentration–time curve, 5 mg per milliliter per minute) on day 1 every 3 weeks for 4 cycles.

### 2.5. Statistical Analysis

Statistical analysis was performed using “The R Foundation for Statistical Computing, R Core Team (2020)”, version 4.0.2. The sensitivity level was 95% with *p* < 0.05, considered statistically significant. In order to assess the continuous variables, we used a Shapiro–Wilk test, and we tested whether we had a Gaussian distribution. If we found that we had a Gaussian distribution, we used a Welch Two Sample *t*-test. For the discrete variables, we used Pearson’s Chi-squared test or Fisher’s exact test. For this study, we determined the OS RMST from the beginning of the treatment with either Nivolumab or Pembrolizumab alone or together with CHT up to 15 May 2022. We calculated the OS RMST for the entire lot, considering the presence or the absence of irAEs and the lines of treatment. We used Cox multivariate proportional analysis model to evaluate the hazard ratio (HR) for a series of parameters: the presence or the absence of irAE, age, sex, type of cancer, type of treatment, stage, ECOG performance status and number of organs affected by metastasis at the initiation of ICIs treatment.

## 3. Results

### 3.1. Baseline Characteristics

The study has seventy-nine patients with a mean age of 63.8 (range between 39 years and 85 years). We enrolled fifty-four (68.4%) male patients and twenty-five (31.6%) females. Sixty-four patients (81%) were in stage IV at diagnosis, and fifteen patients (19%) had stages I to III. Fifty-three patients (67.1%) were treated with Pembrolizumab +/− CHT, and twenty-six patients (32.9%) received Nivolumab as treatment. In patients with CHT and immunotherapy, no cases of febrile neutropenia were reported. Primary prophylaxis of febrile neutropenia was preferred in the presence of intermediate overall risk after considering all the risk factors. Most of the patients had ECOG 0 or ECOG 1 (81.76%), while only ten patients had ECOG 2. With regards to tumor load, most of the patients (86.07%) had one or two organs affected by metastasis. The age, sex, stages, histopathology aspects, ECOG, number of metastatic organs and radiation therapy were not significantly different between the two groups. The administration of chemotherapy had a marginal signification. The only significative difference was the line of the treatment. The results are shown in Table 1.

### 3.2. The Occurrence of irAE

In our study, thirty-four patients (43%) presented irAEs. The irAEs present in the study were endocrine irAEs represented by thyroiditis (15/34; 44.12%) and hypophysis (1/34; 2.94%), cutaneous irAEs (6/34; 17.65%), hepatic irAEs (6/34; 17.65%), renal irAEs (1/34; 2.94%), pulmonary irAEs (2/34; 5.88%), myocardial irAEs (1/34; 2.94%) and rheumatic irAEs (2/34; 5.88%). We observed in our study that 26.47% (9/34) of patients presented multiple irAEs during ICI treatment. The majority were dermatological and endocrine irAEs.

In our study, most patients experienced grade 1 (11/34; 13.92%) and grade 2 (12/34; 15.19%) irAEs. Only 10.13% of patients (8/34) had grade 3 irAEs, and 3.8% of patients (3/34) had grade 4 irAEs. Of the 11 patients who presented with severe irAEs, only 4 continued the treatment, and 7 patients received chemotherapy or palliative care after the remission of their reactions. Of the four patients who continued the treatment, one patient received the treatment in a desensitization protocol, and three patients continued treatment after the remission of the reactions.

### 3.3. The OS RMST

**Figure 2:**
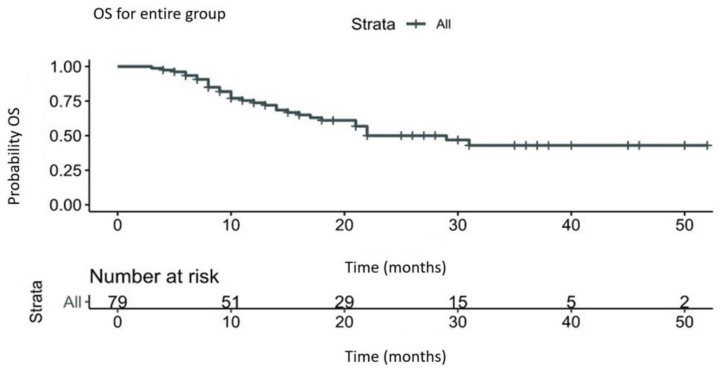
OS RMST for the entire group. Legend: OS—overall survival; RMST—restricted mean survival time.

The OS RMST for the entire group was 30.91 months, with a median survival of 22 months. The events (deaths) for the entire group were 32/79 (40.5%). The results are summarized in Figure 2 and Table 2.

**Figure 3:**
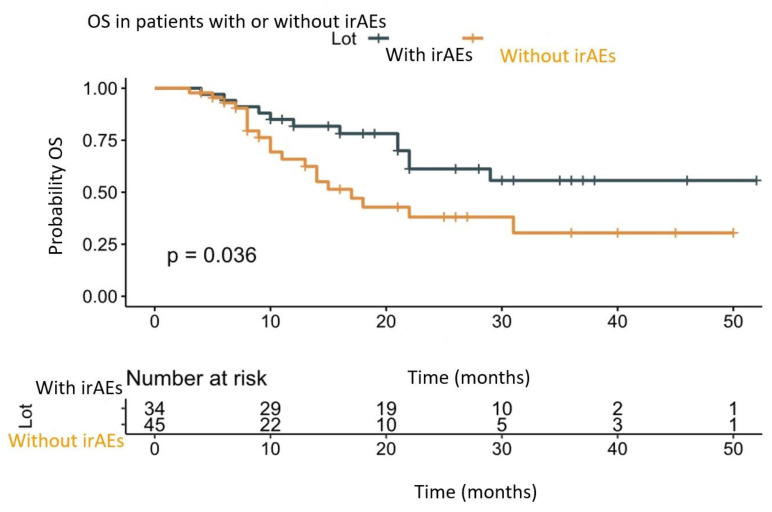
OS in patients with or without irAE. Legend: OS—overall survival; irAEs—immune-related adverse events.

In order to determine if the occurrence of irAEs has a potential role in prolonging OS, we divided the patients into two groups. One group consisted of patients who presented irAEs (*n* = 34), and the other was of patients without irAEs (*n* = 45). In our study, the RMST and events (deaths) percentage were in favor of the patient that presented irAEs (long-rank test, *p* = 0.036). The results are shown in Figure 3.

The OS RMST for the patients with irAEs was 35.7 months, the number of events (deaths) was 12/34 (35.29%), the OS RMST for the patients without irAEs was 17 months and the number of events (deaths) 20/45 (44.44%). The results of this research are shown in Table 3.

Regarding the OS RMST by lines of treatment, patients treated in the first-line settings had an OS RMST of 25.9 months, and patients treated in the second-line settings had an OS RMST of 30.3 months. The results of this research are shown in Table 4.

**Figure 4:**
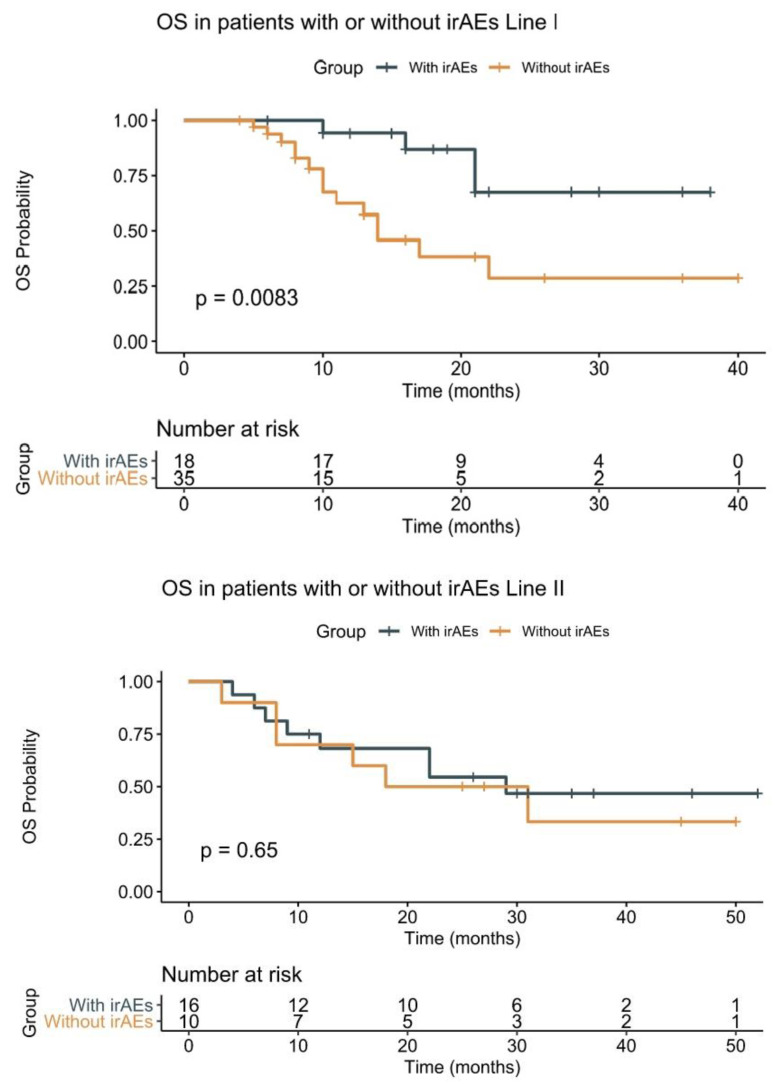
OS RMST by line of treatment. Legend: OS—overall survival; irAEs—immune-related adverse events.

We also estimated OS RMST by lines of treatment and the presence of irAEs. Our study obtained a positive correlation on survival between the presence of irAEs and the first-line treatment (long-rank, *p* = 0.0083) and no significant correlation between the presence of irAEs and the second-line treatment (long-rank, *p* = 0.65). The results are shown in Figure 4. 

We observed in our study that patients with grade 1 and grade 2 had the longest OS RMST, and patients with grades 3 and 4 and without any irAEs had the lowest OS RMST, Table 5.

To estimate the death HR, we analyzed the independent predictors of the death event. In our research, the risk of dying was 2.13 times higher for the patients without irAEs than for patients who presented irAEs, with a confidence interval (CI) of 95% of 1.03 to 4.39. Moreover, increasing ECOG performance status by one point increased the risk of death by 9.52 times, with a CI 95% of 2.71 to 33.6, and the involvement of more metastatic organs was associated with a 1.60 times increase in the risk of death, with a CI 95% of 1.09 to 2.36. In our study, males had an increased risk of death by 2.21 times higher than females, with a CI 95% of 0.91 to 5.38, and smoking status raised the risk of death by 0.47 with a CI 95% of 0.19 to 1.14, but without statistical significance. In the Cox simple analysis, age, the type of tumor and the chemotherapy were not seen as predictors for the death. The results are summarized in Table 6.

After adjustment for age, sex, smoking status, type of tumor, ECOG performance status and chemotherapy administration, in the Cox multivariate analysis, the presence of irAEs continued to be a predictor of death by 2.59, with a CI of 1.10 to 6.08 and ECOG performance status increased by one point raised the risk of death by 3.77 points. The age, sex, smoking status, type of tumor and chemotherapy were not seen as predictors for death. The results are shown in Table 7.

## 4. Discussion

In our study, we investigated whether RMST could be used as a tool for the survival of patients treated with ICIs and whether the presence of irAEs plays a potential role in the survival of these patients.

RMST analysis is an endpoint that can replace HR. In studies with immunotherapy, the Cox proportional hazard, which assumes that the hazard rate is constant throughout the entire follow-up of the treatment group and of the control group, is constantly broken. This violation of the PH assumption is observed in RCT [23,25]. Our study shows a violation of PH up to 10 months on survival curves in Figure 2.

In the updated analysis of Checkmate 017 and 057, after 5 years of follow-ups, the median duration of therapy was 36.9 months, and 18 out of 50 patients remained on Nivolumab > 5 years. In the Nivolumab group, 10% of the patients were without treatment after a median duration of treatment of 41.9 months [30]. In the updated analysis of KEYNOTE 189, the median of OS was 22 months, with a median follow-up of 23.1 months [31]. In our study, the OS RMST for the entire group was 30.91 months, and for the patients with irAEs was 35.7 months. This is the first study that has evaluated and reported the OS RMST for patients with irAEs, although we have included patients in the first- and second-line settings. We analyzed and concluded that patients treated in the first-line settings that presented irAEs had a significant OS RMST (31.5 months vs. 20.4 months, *p* = 0.0083) over patients without irAEs. For patients treated in the second-line settings, OS RMST was not significant in the two subgroups (32 months vs. 27 months, *p* = 0.65). A possible explanation for this fact is the number of patients in the second-line setting, as we only included 26 patients.

We know from the studies that the presence of irAEs has a controversial effect on survival. Haratani K. et al. demonstrated in a study that the development of irAEs is associated with better responses in patients with LC treated with Nivolumab [32]. In another study, Owen DH. et al. showed that survival is not correlated with the development of irAEs, but the patients who developed irAEs of the thyroid had a longer survival compared with those who did not [33]. In a recent extensive meta-analysis, Zhong et al. showed that the patients with irAEs had better survival than those without irAEs. The presence of cutaneous, endocrine and gastrointestinal irAEs was associated with better outcomes. Regarding the grade of the irAE, patients with a low grade of the irAEs had an improved OS [34]. Although the number of patients was low, our research demonstrated that patients with irAEs had better survival compared with those without irAEs (35.7 months vs. 17 months, *p* = 0.036). We also analyzed whether the grade of irAEs influences the survival, and we found that patients with grade 1 and grade 2 had the longest survivals (28.2 months and 34.1 months), and the patients with grade 3 and 4 had the lowest survivals (26.5 months). These results have to be cautiously regarded because of the small number of patients stratified according to the grades of irAEs but are comparable with the results reported in the literature [34]. The incidence of the irAEs in our study was 43.04% comparable with other studies in the literature [32,33,34,35].

Among sex, age, weight loss, tumor type and markers of systemic inflammation, ECOG performance status is known as a prognostic factor in LC [36]. When using the international guidelines, patients with ECOG 0, 1 and 2 are considered fit for treatment with CHT, while patients with ECOG 3 and 4 are candidates for palliative treatments. In more recent years, researchers tried to identify if patients with ECOG 3 or 4 can benefit from treatment with ICIs. The results found are controversial in the literature. Sehgal K. et al. sustained that those patients with an ECOG of at least two had a poor outcome when they were treated with ICIs in monotherapy [37]. Kapoor A. et al. reported that treatment with ICIs is effective even in patients with poor performance status. Survival at 12 months, in his study, was 35.4% for ECOG 2 and 17.7% for ECOG 3–4 [38]. Mojsak D. et al. also supported that treatment with ICIs in patients with ECOG 2 is feasible, but the survival is insignificant for this group [39]. However, our study demonstrated that patients with a poor ECOG performance status have an increased risk of death.

The systemic tumor burden of LC is reflected by the number of metastatic organs. We know from the studies that LC patients with brain, bone and liver metastases have a poor prognostic [40]. There are only a few studies in the literature that have evaluated whether the number of metastatic organs is correlated with the survival benefits or the assessment of Ki 67 [41,42,43,44]. In our study, which is consistent with the literature, we found that patients who presented at the initiation of ICI with more than two metastatic organs had poor outcomes. We have to bear in mind the combination of ICIs and antibiotics or antiretroviral therapy since their interactions can decrease the efficacy of ICIs treatment [45,46,47,48].

Smoking status has an important role in patients receiving treatment with ICIs. The studies from the literature demonstrated that patients that received ICIs in the first line of treatment and were former or current smokers had survival advantages because smoking is associated with high tumor mutational burden (TMB). In ICIs studies, TMB is known as a predictive biomarker for responses to ICIs treatment [49,50]. In our study, the status of current or former status did not translate into a survival advantage.

The limitations of our study are represented by the small number of patients and the treatment type (first and second-line of treatment) used. Our findings need to be evaluated in larger prospective studies.

## 5. Conclusions

The RMST is a new tool that may help the researchers to better address the survival in studies with ICIs treatment where PH fails and the long-rank test is less efficient due to the existence of the long-term responses and delayed treatment effects. As the number of patients is small in our study, it is hypothesized that patients that are offered ICIs treatment in the first-line settings and develop irAEs have a better survival rate than those without irAEs. ECOG performance status and the number of metastatic organs can help clinicians to better select their patients for treatment with ICIs.

## Figures and Tables

**Table 1:** The variables used in the study.

Variables	With irAEs, *N* = 34 ^1^	Without irAEs, *N* = 45 ^1^	*p*-Value ^2^
Age	64.41 (10.03)	63.40 (9.46)	0.650
Sex			0.906
F	11 (32%)	14 (31%)	
M	23 (68%)	31 (69%)	
ECOG			0.293
0	9 (26.5%)	12 (27%)	
1	23 (67.6 %)	25 (56%)	
2	2 (5.9 %)	8 (17%)	
Stage at diagnosis			0.400
I–III	5 (15%)	10 (22%)	
IV	29 (85%)	35 (78%)	
Histopathology			0.486
ADK	25 (74%)	31 (69%)	
Squamous	8 (24%)	9 (20%)	
Other types	1 (2.9%)	5 (11%)	
Patient with chemotherapy			0.088
Yes	17 (50%)	14 (31.1%)	
No	17 (50%)	31 (68.9%)	
Patient with radiation therapy			0.351
Yes	9 (26.4%)	8 (17.7%)	
No	25 (73.6%)	37 (82.3%)	
Treatment line			0.020
I	18 (53%)	35 (78%)	
II	16 (47%)	10 (22%)	
Number of organs affected by metastasis	1.74 (0.67)	1.76 (0.71)	0.861

Legend: irAEs—immune-related adverse effects; ADK—adenocarcinoma; CHT—chemotherapy; ECOG—performance status. ^1^ Mean (SD); *n* (%); ^2^ Welch Two Sample *t*-test; Pearson’s Chi-squared test; Fisher’s exact test.

**Table 2:** Restricted mean survival time and number of events (deaths) for the entire group.

Strata	*N* Deaths (%)	RMST	Median Survival (95% CI)
**Entire Group**	32/79 (40.50)	30.91	22.00 [18.00 la N/A]

Legend: RMST—Restricted mean survival time, CI—confidence interval, N/A-not applicable.

**Table 3:** OS in patients with or without irAEs.

Strata	*N* Deaths (%)	RMST	Median Survival (95% CI)
**With irAEs**	12/34 (35.29)	35.70	N/A [22.00 la N/A]
**Without irAEs**	20/45 (44.44)	17.00	17.00 [13.00 la N/A]

Legend: irAEs—immune-related adverse events; RMST—restricted mean survival time; CI—confidence interval; N/A—not applicable.

**Table 4:** OS in patients treated in the first- and second-line settings.

Strata	*N* Deaths (%)	RMST	Median Survival (95% CI)
**First line treatment**	18/53 (33.96)	25.90	22.00 [16.00 la N/A]
**Second line treatment**	14/26 (53.84)	30.30	29.00 [15.00 la N/A]

Legend: RMST—restricted mean survival time; CI—confidence interval; N/A—not applicable.

**Table 5:** OS RMST according to the grades of irAEs.

Strata Grade of irAEs	*N* Deaths (%)	RMST	Median Survival (95% CI)
**Without irAEs**	20/45 (44.44)	24.90	17.00 (13.00 la N/A)
**Grade 1**	2/11 (18.18)	28.20	N/A (29.00 la N/A)
**Grade 2**	5/12 (41.66)	34.10	N/A (16.00 la N/A)
**Grade 3 and 4**	5/11 (45.45)	26.50	22.00 (21.00 la N/A)

Legend: irAEs—immune-related adverse events; RMST—restricted mean survival time; CI—confidence interval; N/A—not applicable.

**Table 6:** Cox simple regression.

Predictor	*N*	Event *N*	HR (CI 95%) ^1^	*p*-Value
Groups				
With irAEs	34	12	—	
Without irAEs	45	20	2.13 (1.03 to 4.39)	0.041
Age	79	32	0.99 (0.96 to 1.03)	0.690
Sex				
F	25	6	—	
M	54	26	2.21 (0.91 to 5.38)	0.080
Smoking status				
Yes	52	24	—	
No	23	6	0.47 (0.19 to 1.14)	0.096
Type of tumor				
ADK	56	21	—	
Squamous	17	9	1.92 (0.87 to 4.27)	0.108
Other types	6	2	0.79 (0.18 to 3.41)	0.753
ECOG performance status				
0	21	4	—	
1	48	20	2.71 (0.92 to 7.97)	0.070
2	10	8	9.55 (2.71 to 33.6)	<0.001
Patients with chemotherapy				
Yes	31	15	—	
No	48	17	0.90 (0.44 to 1.82)	0.764
Number of organs affected by metastasis	53	32	1.60 (1.09 to 2.36)	0.018

Legend: irAEs—immune-related adverse events; ADK—adenocarcinoma; HR—hazard ratio; CI—confidence interval; ECOG performance status—Eastern Cooperative Oncology Group performance status. ^1^ HR = Hazard Ratio, CI = Confidence Interval.

**Table 7:** Cox multivariate analyses.

Predictor	*N*	Event *N*	HR (CI 95%) ^1^	*p*-Value
Groups				
With irAEs	31	10	—	
Without irAEs	44	20	2.59 (1.10 to 6.08)	0.029
Age	75	30	0.99 (0.95 to 1.04)	0.80
Sex				
F	25	6	—	
M	50	24	1.49 (0.52 to 4.27)	0.46
Smoking status				
Yes	52	24	—	
No	23	6	0.71 (0.26 to 1.95)	0.51
Type of tumor				
ADK	54	19	—	
Squamous	15	9	1.90 (0.81 to 4.48)	0.14
Other types	6	2	0.57 (0.10 to 3.14)	0.52
ECOG performance status				
0	21	4	—	
1	44	18	2.92 (0.92 to 9.20)	0.068
2	10	8	6.69 (1.71 to 26.2)	0.006
Patients with chemotherapy				
Yes	30	14	—	
No	45	16	0.67 (0.31 to 1.45)	0.31

Legend: irAEs—immune-related adverse events; ADK—adenocarcinoma; HR—hazard ratio; CI—confidence interval; ECOG performance status—Eastern Cooperative Oncology Group performance status. ^1^ HR = Hazard Ratio, CI = Confidence Interval.

## Data Availability

Not applicable.
